# *catena*-Poly[[triaqua­(pyridine-κ*N*)nickel(II)]-μ-sulfato-κ^2^ *O*:*O*′]

**DOI:** 10.1107/S1600536809049605

**Published:** 2009-11-25

**Authors:** Yan-Fang Shi, Fu-Xing Li, Bo Geng, Yan-Cheng Liu, Zhen-Feng Chen

**Affiliations:** a Key Laboratory for the Chemistry and Molecular Engineering of Medicinal Resources (Ministry of Education of China), School of Chemistry & Chemical Engineering, Guangxi Normal University, Guilin 541004, People’s Republic of China

## Abstract

The title compound, [Ni(SO_4_)(C_5_H_5_N)(H_2_O)_3_]_*n*_, was synthesized by the hydro­thermal reaction of NiSO_4_·6H_2_O, pyridine and water. The central Ni^II^ atom is coordinated in a distorted octa­hedral environment by a pyridine N atom, three aqua O atoms and two O atoms of bridging sulfate anions, yielding a zigzag chain. A three-dimensional network is generated *via* complex hydrogen bonds involving the sulfate and aqua ligands and a pyridine C—H group.

## Related literature

For the structures of related nickel(II) complexes, see: Wang *et al.* (2006 ▶); Stein *et al.* (2007 ▶).
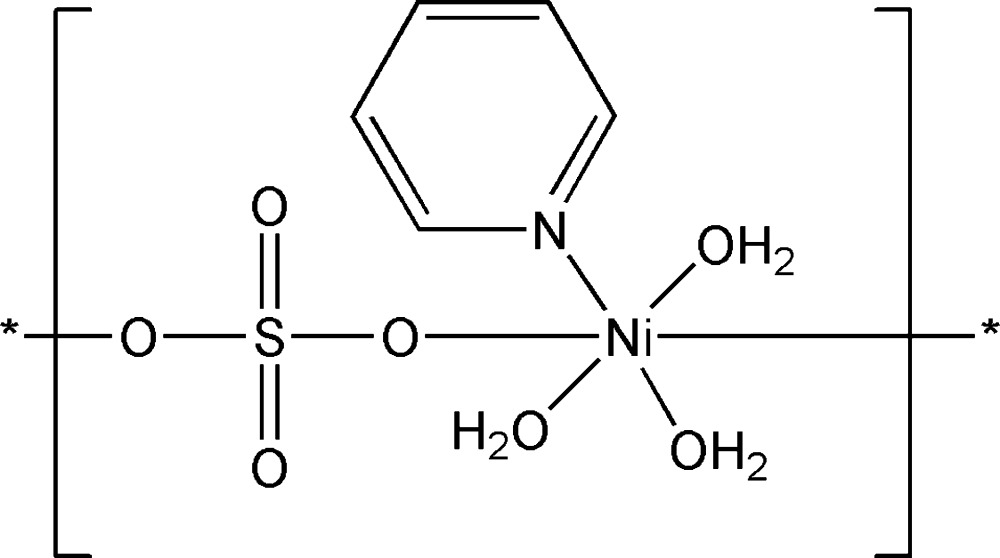


## Experimental

### 

#### Crystal data

[Ni(SO_4_)(C_5_H_5_N)(H_2_O)_3_]
*M* *_r_* = 287.92
Monoclinic, 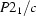

*a* = 11.868 (3) Å
*b* = 7.5745 (14) Å
*c* = 11.420 (3) Å
β = 110.724 (4)°
*V* = 960.2 (3) Å^3^
*Z* = 4
Mo *K*α radiation
μ = 2.26 mm^−1^
*T* = 193 K
0.30 × 0.20 × 0.14 mm

#### Data collection

Rigaku Mercury CCD diffractometer
Absorption correction: multi-scan (*REQAB*; Jacobson, 1998 ▶) *T*_min_ = 0.465, *T*_max_ = 0.729
8854 measured reflections
1746 independent reflections
1641 reflections with *I* > 2σ(*I*)
*R*_int_ = 0.040

#### Refinement

*R*[*F* ^2^ > 2σ(*F* ^2^)] = 0.036
*wR*(*F* ^2^) = 0.110
*S* = 1.16
1746 reflections
161 parameters
6 restraints
H atoms treated by a mixture of independent and constrained refinement
Δρ_max_ = 0.57 e Å^−3^
Δρ_min_ = −0.84 e Å^−3^

### 

Data collection: *CrystalClear* (Rigaku, 1999 ▶); cell refinement: *CrystalClear*; data reduction: *CrystalStructure* (Rigaku/MSC & Rigaku, 2000 ▶); program(s) used to solve structure: *SHELXS97* (Sheldrick, 2008 ▶); program(s) used to refine structure: *SHELXL97* (Sheldrick, 2008 ▶); molecular graphics: *SHELXTL* (Sheldrick, 2008 ▶); software used to prepare material for publication: *SHELXTL*.

## Supplementary Material

Crystal structure: contains datablocks I, global. DOI: 10.1107/S1600536809049605/pk2206sup1.cif

Structure factors: contains datablocks I. DOI: 10.1107/S1600536809049605/pk2206Isup2.hkl

Additional supplementary materials:  crystallographic information; 3D view; checkCIF report

## Figures and Tables

**Table 1:** Hydrogen-bond geometry (Å, °)

*D*—H⋯*A*	*D*—H	H⋯*A*	*D*⋯*A*	*D*—H⋯*A*
O5—H5*A*⋯O4^i^	0.82 (3)	2.04 (3)	2.849 (3)	170 (4)
O5—H5*B*⋯O1^ii^	0.818 (10)	1.939 (12)	2.753 (3)	173 (4)
O6—H6*A*⋯O3^i^	0.821 (10)	1.949 (12)	2.764 (3)	172 (4)
O6—H6*B*⋯O4	0.82 (3)	2.15 (3)	2.821 (3)	139 (4)
O7—H7*A*⋯O2^ii^	0.82 (3)	2.00 (3)	2.817 (3)	176 (4)
O7—H7*B*⋯O4^iii^	0.815 (10)	1.94 (2)	2.690 (3)	153 (4)
C4—H4⋯O3^iv^	0.95	2.57	3.304 (5)	135

Symmetry codes: (i) 
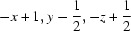
; (ii) 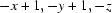; (iii) 
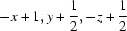
; (iv) 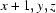.

**Fig. 1.**
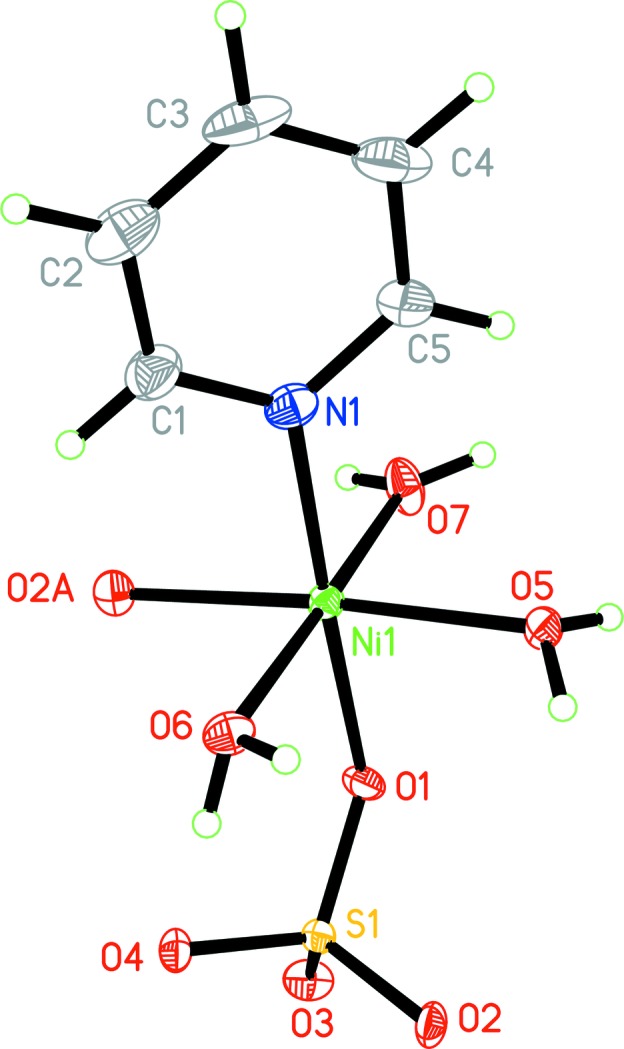
The molecular structure showing the atom-labelling scheme. Displacement ellipsoids are drawn at the 50% probability level.

**Fig. 2.**
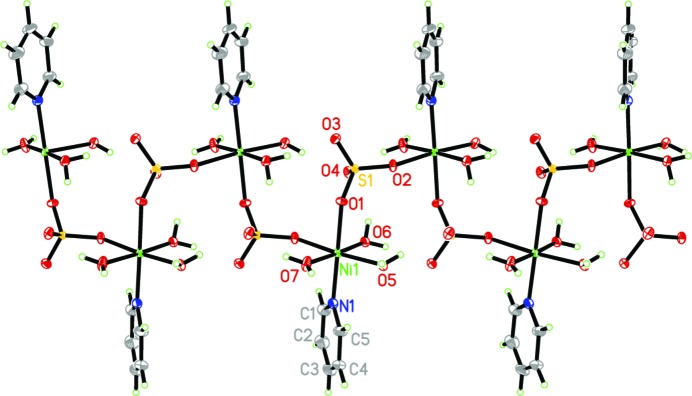
A view of the one-dimensional chain structure that propagates along the b axis.

**Fig. 3.**
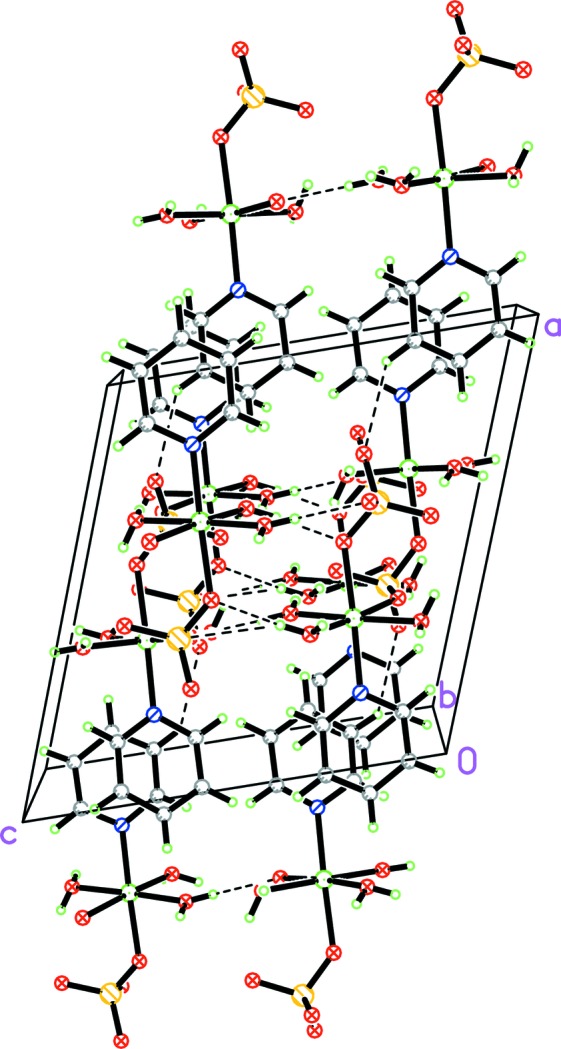
A packing diagram viewed approximately down the b axis.

## supplementary crystallographic information

### Comment

The asymmetric unit contains one independent Ni atom, which is octahedrally coordinated by two sulfato anions, three aqua ligands and one pyridine molecule. The bond lengths and angles involving Ni—*O*(aqua), Ni—N are similar to those of other nickel-carboxylate coordination polymers with pyridine (Wang *et al.*, 2006; Stein *et al.*, 2007), with the Ni center displaying the typical distorted octahedral coordination, which can be viewed from the angles of N1—Ni1—O1 177.81 (10)°, N1—Ni1—O7 91.13 (11)°, O1—Ni1—O6 92.13 (9)°, O5—Ni1—O6 92.91 (10)° (Fig. 1). The SO_4_^2-^ dianion acts as a µ_2_ bridging ligand, linking two adjacent metal ions and generating a one-dimensional zigzag chain (Fig. 2). The aqua ligands, sulfato groups and C—H of pyridine form extensive hydrogen-bonding interactions (Table 1), resulting in a three-dimensional network (Fig. 3).

### Experimental

Samples of NiSO_4_.6H_2_O (0.1 mmol) and pyridine (0.1 mmol) were placed in a thick-walled Pyrex tube (*ca* 20 cm long). After addition of H_2_O (1 ml), the tube was frozen with liquid nitrogen, evacuated under vacuum and sealed with a torch. The tube was heated at 110°C for 2 days and then was slowly cooled down to room temperature, and light-green block-shaped crystals were obtained. Yield: 35%.

### Refinement

The H atoms bonded to C atoms were positioned geometrically and refined using a riding model with *U*_iso_(H) = 1.2*U*_eq_(C) (C—H = 0.95 Å). Water H positions were located in an electron-density difference map and refined freely.

### Crystal data

**Table:**

[Ni(SO_4_)(C_5_H_5_N)(H_2_O)_3_]	*F*(000) = 592
*M**_r_* = 287.92	*D*_x_ = 1.992 Mg m^−^^3^
Monoclinic, *P*2_1_/*c*	Mo *K*α radiation, λ = 0.71070 Å
Hall symbol: -P 2ybc	Cell parameters from 3550 reflections
*a* = 11.868 (3) Å	θ = 3.1–25.3°
*b* = 7.5745 (14) Å	µ = 2.26 mm^−^^1^
*c* = 11.420 (3) Å	*T* = 193 K
β = 110.724 (4)°	Block, light-green
*V* = 960.2 (3) Å^3^	0.30 × 0.20 × 0.14 mm
*Z* = 4	

### Data collection

**Table:**

Rigaku Mercury CCD diffractometer	1746 independent reflections
Radiation source: fine-focus sealed tube	1641 reflections with *I* > 2σ(*I*)
graphite	*R*_int_ = 0.040
Detector resolution: 7.31 pixels mm^-1^	θ_max_ = 25.3°, θ_min_ = 3.3°
ω scans	*h* = −14→13
Absorption correction: multi-scan (REQAB; Jacobson, 1998)	*k* = −9→9
*T*_min_ = 0.465, *T*_max_ = 0.729	*l* = −13→12
8854 measured reflections	

### Refinement

**Table:**

Refinement on *F*^2^	Primary atom site location: structure-invariant direct methods
Least-squares matrix: full	Secondary atom site location: difference Fourier map
*R*[*F*^2^ > 2σ(*F*^2^)] = 0.036	Hydrogen site location: inferred from neighbouring sites
*wR*(*F*^2^) = 0.110	H atoms treated by a mixture of independent and constrained refinement
*S* = 1.16	*w* = 1/[σ^2^(*F*_o_^2^) + (0.0672*P*)^2^ + 0.4274*P*] where *P* = (*F*_o_^2^ + 2*F*_c_^2^)/3
1746 reflections	(Δ/σ)_max_ = 0.001
161 parameters	Δρ_max_ = 0.57 e Å^−^^3^
6 restraints	Δρ_min_ = −0.84 e Å^−^^3^

### Special details

**Table:**

Geometry. All e.s.d.'s (except the e.s.d. in the dihedral angle between two l.s. planes) are estimated using the full covariance matrix. The cell e.s.d.'s are taken into account individually in the estimation of e.s.d.'s in distances, angles and torsion angles; correlations between e.s.d.'s in cell parameters are only used when they are defined by crystal symmetry. An approximate (isotropic) treatment of cell e.s.d.'s is used for estimating e.s.d.'s involving l.s. planes.
Refinement. Refinement of *F*^2^ against ALL reflections. The weighted *R*-factor *wR* and goodness of fit *S* are based on *F*^2^, conventional *R*-factors *R* are based on *F*, with *F* set to zero for negative *F*^2^. The threshold expression of *F*^2^ > σ(*F*^2^) is used only for calculating *R*-factors(gt) *etc*. and is not relevant to the choice of reflections for refinement. *R*-factors based on *F*^2^ are statistically about twice as large as those based on *F*, and *R*- factors based on ALL data will be even larger.

### Fractional atomic coordinates and isotropic or equivalent isotropic displacement parameters (Å^2^)

**Table:**

	*x*	*y*	*z*	*U*_iso_*/*U*_eq_	
Ni1	0.65136 (3)	0.59664 (5)	0.21049 (4)	0.0112 (2)	
S1	0.37812 (7)	0.51034 (10)	0.20733 (7)	0.0111 (2)	
O1	0.4632 (2)	0.5757 (3)	0.1473 (2)	0.0144 (5)	
O2	0.3600 (2)	0.3178 (3)	0.1818 (2)	0.0148 (5)	
O3	0.2651 (2)	0.6067 (3)	0.1535 (2)	0.0185 (6)	
O4	0.4322 (2)	0.5392 (3)	0.3441 (2)	0.0163 (5)	
O5	0.6504 (2)	0.3741 (3)	0.1062 (2)	0.0139 (5)	
H5A	0.626 (3)	0.283 (3)	0.128 (3)	0.017 (10)*	
H5B	0.619 (4)	0.381 (6)	0.0300 (11)	0.033 (13)*	
O6	0.6708 (2)	0.4525 (3)	0.3691 (2)	0.0165 (5)	
H6A	0.687 (4)	0.3472 (19)	0.367 (4)	0.037 (13)*	
H6B	0.611 (2)	0.435 (5)	0.387 (4)	0.028 (12)*	
O7	0.6283 (2)	0.7484 (3)	0.0563 (2)	0.0212 (6)	
H7A	0.632 (4)	0.724 (5)	−0.0120 (19)	0.034 (12)*	
H7B	0.609 (4)	0.850 (2)	0.064 (4)	0.031 (12)*	
N1	0.8352 (3)	0.6199 (4)	0.2656 (3)	0.0179 (7)	
C1	0.9028 (3)	0.6672 (5)	0.3822 (3)	0.0247 (8)	
H1	0.8637	0.6950	0.4394	0.030*	
C2	1.0269 (3)	0.6774 (6)	0.4232 (4)	0.0325 (9)	
H2	1.0718	0.7112	0.5069	0.039*	
C3	1.0846 (3)	0.6379 (6)	0.3407 (4)	0.0345 (10)	
H3	1.1699	0.6441	0.3664	0.041*	
C4	1.0165 (4)	0.5894 (5)	0.2207 (5)	0.0331 (10)	
H4	1.0539	0.5612	0.1620	0.040*	
C5	0.8929 (3)	0.5822 (5)	0.1867 (4)	0.0255 (9)	
H5C	0.8464	0.5490	0.1034	0.031*	

### Atomic displacement parameters (Å^2^)

**Table:**

	*U*^11^	*U*^22^	*U*^33^	*U*^12^	*U*^13^	*U*^23^
Ni1	0.0115 (3)	0.0107 (3)	0.0109 (3)	−0.00030 (14)	0.0035 (2)	0.00009 (15)
S1	0.0122 (4)	0.0103 (4)	0.0114 (4)	−0.0005 (3)	0.0050 (3)	−0.0009 (3)
O1	0.0123 (12)	0.0198 (13)	0.0129 (12)	−0.0013 (9)	0.0066 (10)	0.0014 (9)
O2	0.0222 (13)	0.0097 (12)	0.0127 (11)	−0.0012 (9)	0.0064 (10)	−0.0036 (10)
O3	0.0172 (13)	0.0147 (13)	0.0259 (14)	0.0034 (9)	0.0103 (11)	0.0035 (10)
O4	0.0226 (13)	0.0160 (12)	0.0113 (12)	−0.0048 (10)	0.0071 (10)	−0.0043 (10)
O5	0.0188 (13)	0.0144 (12)	0.0081 (12)	−0.0028 (10)	0.0042 (10)	0.0010 (10)
O6	0.0158 (13)	0.0135 (13)	0.0198 (13)	−0.0005 (11)	0.0058 (10)	0.0022 (11)
O7	0.0361 (15)	0.0135 (14)	0.0175 (14)	0.0053 (11)	0.0139 (12)	0.0009 (11)
N1	0.0154 (15)	0.0150 (15)	0.0223 (16)	−0.0002 (11)	0.0053 (12)	0.0016 (12)
C1	0.0198 (19)	0.029 (2)	0.0217 (19)	−0.0036 (16)	0.0025 (15)	−0.0012 (17)
C2	0.020 (2)	0.035 (2)	0.033 (2)	−0.0033 (17)	−0.0025 (17)	0.002 (2)
C3	0.0139 (19)	0.028 (2)	0.057 (3)	−0.0045 (16)	0.007 (2)	0.004 (2)
C4	0.024 (2)	0.028 (2)	0.056 (3)	−0.0004 (17)	0.025 (2)	0.002 (2)
C5	0.0198 (19)	0.029 (2)	0.030 (2)	−0.0035 (15)	0.0124 (17)	−0.0038 (17)

### Geometric parameters (Å, °)

**Table:**

Ni1—O7	2.039 (2)	O6—H6B	0.82 (3)
Ni1—N1	2.053 (3)	O7—H7A	0.82 (3)
Ni1—O6	2.056 (2)	O7—H7B	0.815 (10)
Ni1—O5	2.062 (2)	N1—C1	1.337 (5)
Ni1—O1	2.096 (2)	N1—C5	1.342 (5)
Ni1—O2^i^	2.110 (2)	C1—C2	1.380 (5)
S1—O3	1.458 (2)	C1—H1	0.9500
S1—O4	1.479 (2)	C2—C3	1.380 (6)
S1—O2	1.488 (2)	C2—H2	0.9500
S1—O1	1.491 (2)	C3—C4	1.372 (6)
O2—Ni1^ii^	2.110 (2)	C3—H3	0.9500
O5—H5A	0.82 (3)	C4—C5	1.379 (6)
O5—H5B	0.818 (10)	C4—H4	0.9500
O6—H6A	0.821 (10)	C5—H5C	0.9500
			
O7—Ni1—N1	91.13 (11)	H5A—O5—H5B	108 (4)
O7—Ni1—O6	177.36 (10)	Ni1—O6—H6A	117 (3)
N1—Ni1—O6	89.97 (11)	Ni1—O6—H6B	118 (3)
O7—Ni1—O5	89.45 (9)	H6A—O6—H6B	94 (4)
N1—Ni1—O5	92.04 (11)	Ni1—O7—H7A	131 (3)
O6—Ni1—O5	92.91 (10)	Ni1—O7—H7B	113 (3)
O7—Ni1—O1	86.80 (10)	H7A—O7—H7B	115 (4)
N1—Ni1—O1	177.81 (10)	C1—N1—C5	117.1 (3)
O6—Ni1—O1	92.13 (9)	C1—N1—Ni1	121.8 (2)
O5—Ni1—O1	87.23 (9)	C5—N1—Ni1	121.1 (3)
O7—Ni1—O2^i^	92.22 (9)	N1—C1—C2	123.1 (4)
N1—Ni1—O2^i^	91.89 (10)	N1—C1—H1	118.5
O6—Ni1—O2^i^	85.33 (9)	C2—C1—H1	118.5
O5—Ni1—O2^i^	175.69 (9)	C3—C2—C1	118.9 (4)
O1—Ni1—O2^i^	88.90 (9)	C3—C2—H2	120.5
O3—S1—O4	111.21 (14)	C1—C2—H2	120.5
O3—S1—O2	111.13 (13)	C4—C3—C2	118.8 (4)
O4—S1—O2	109.26 (13)	C4—C3—H3	120.6
O3—S1—O1	108.13 (14)	C2—C3—H3	120.6
O4—S1—O1	108.95 (13)	C3—C4—C5	118.9 (4)
O2—S1—O1	108.08 (12)	C3—C4—H4	120.6
S1—O1—Ni1	132.76 (14)	C5—C4—H4	120.6
S1—O2—Ni1^ii^	134.32 (13)	N1—C5—C4	123.2 (4)
Ni1—O5—H5A	116 (3)	N1—C5—H5C	118.4
Ni1—O5—H5B	118 (3)	C4—C5—H5C	118.4
			
O3—S1—O1—Ni1	150.13 (18)	O2^i^—Ni1—N1—C1	37.9 (3)
O4—S1—O1—Ni1	29.1 (2)	O7—Ni1—N1—C5	−52.1 (3)
O2—S1—O1—Ni1	−89.5 (2)	O6—Ni1—N1—C5	130.3 (3)
O7—Ni1—O1—S1	−167.8 (2)	O5—Ni1—N1—C5	37.4 (3)
O6—Ni1—O1—S1	9.8 (2)	O2^i^—Ni1—N1—C5	−144.4 (3)
O5—Ni1—O1—S1	102.64 (19)	C5—N1—C1—C2	−0.3 (5)
O2^i^—Ni1—O1—S1	−75.47 (19)	Ni1—N1—C1—C2	177.5 (3)
O3—S1—O2—Ni1^ii^	−103.2 (2)	N1—C1—C2—C3	0.2 (6)
O4—S1—O2—Ni1^ii^	19.9 (2)	C1—C2—C3—C4	−0.1 (6)
O1—S1—O2—Ni1^ii^	138.29 (18)	C2—C3—C4—C5	0.1 (6)
O7—Ni1—N1—C1	130.2 (3)	C1—N1—C5—C4	0.3 (5)
O6—Ni1—N1—C1	−47.4 (3)	Ni1—N1—C5—C4	−177.5 (3)
O5—Ni1—N1—C1	−140.3 (3)	C3—C4—C5—N1	−0.3 (6)

Symmetry codes: (i) −*x*+1, *y*+1/2, −*z*+1/2; (ii) −*x*+1, *y*−1/2, −*z*+1/2.

### Hydrogen-bond geometry (Å, °)

**Table:**

*D*—H···*A*	*D*—H	H···*A*	*D*···*A*	*D*—H···*A*
O5—H5A···O4^ii^	0.82 (3)	2.04 (3)	2.849 (3)	170 (4)
O5—H5A···S1^ii^	0.82 (3)	2.81 (2)	3.571 (3)	157 (3)
O5—H5B···O1^iii^	0.82 (1)	1.94 (1)	2.753 (3)	173 (4)
O5—H5B···S1^iii^	0.82 (1)	2.85 (2)	3.584 (3)	151 (4)
O6—H6A···O3^ii^	0.82 (1)	1.95 (1)	2.764 (3)	172 (4)
O6—H6A···S1^ii^	0.82 (1)	2.71 (2)	3.458 (3)	152 (4)
O6—H6B···O4	0.82 (3)	2.15 (3)	2.821 (3)	139 (4)
O6—H6B···S1	0.82 (3)	2.85 (4)	3.336 (3)	120 (3)
O7—H7A···O2^iii^	0.82 (3)	2.00 (3)	2.817 (3)	176 (4)
O7—H7A···S1^iii^	0.82 (3)	2.82 (2)	3.571 (3)	154 (4)
O7—H7B···O4^i^	0.82 (1)	1.94 (2)	2.690 (3)	153 (4)
O7—H7B···S1^i^	0.82 (1)	2.84 (4)	3.372 (3)	125 (4)
C4—H4···O3^iv^	0.95	2.57	3.304 (5)	135

Symmetry codes: (ii) −*x*+1, *y*−1/2, −*z*+1/2; (iii) −*x*+1, −*y*+1, −*z*; (i) −*x*+1, *y*+1/2, −*z*+1/2; (iv) *x*+1, *y*, *z*.
